# Methylprednisolone inhibits IFN-γ and IL-17 expression and production by cells infiltrating central nervous system in experimental autoimmune encephalomyelitis

**DOI:** 10.1186/1742-2094-6-37

**Published:** 2009-12-11

**Authors:** Željka Miljković, Miljana Momčilović, Djordje Miljković, Marija Mostarica-Stojković

**Affiliations:** 1 Institute of Microbiology and Immunology, School of Medicine, University of Belgrade, Belgrade, Serbia; 2 Department of Immunology, Institute for Biological Research "Siniša Stanković", University of Belgrade, Belgrade, Serbia

## Abstract

**Background:**

Glucocorticoids have been shown to be effective in the treatment of autoimmune diseases of the CNS such as multiple sclerosis and its animal model, experimental autoimmune encephalomyelitis (EAE). However, the mechanisms and the site of glucocorticoids' actions are still not completely defined. The aim of this study was to investigate the *in vivo *effect of the synthetic glucocorticoid methylprednisolone (MP) on the expression and production of proinflammatory cytokines interferon (IFN)-γ and interleukin (IL)-17 by cells infiltrating CNS tissue.

**Methods:**

Experimental autoimmune encephalomyelitis was induced in Dark Agouti (DA) rats by immunization with rat spinal cord homogenate mixed with adjuvants. Commencing on the day when the first EAE signs appeared, DA rats were injected daily for 3 days with MP and/or RU486, an antagonist of glucocorticoid receptor. Cytokine production and gene expression in CNS-infiltrating cells and lymph node cells were measured using ELISA and real time PCR, respectively.

**Results:**

Treatment of rats with MP ameliorated EAE, and the animals recovered without relapses. Further, MP inhibited IFN-γ and IL-17 expression and production in cells isolated from the CNS of DA rats with EAE after the last injection of MP. The observed effect of MP *in vivo *treatment was not mediated through depletion of CD4^+ ^T cells among CNS infiltrating cells, or through induction of their apoptosis within the CNS. Finally, the glucocorticoid receptor-antagonist RU486 prevented the inhibitory effect of MP on IFN-γ and IL-17 production both *in vitro *and *in vivo*, thus indicating that the observed effects of MP were mediated through glucocorticoid receptor-dependent mechanisms.

**Conclusion:**

Taken together, these results demonstrate that amelioration of EAE by exogenous glucocorticoids might be, at least partly, ascribed to the limitation of effector cell functions in the target tissue.

## Background

Multiple sclerosis (MS) is a chronic, inflammatory, demyelinating disease of the CNS with a putative autoimmune pathogenesis [1]. Its widely used model is experimental autoimmune encephalomyelitis (EAE), an organ-specific autoimmune inflammatory disease induced in susceptible animals, shows significant similarities to MS in both clinical and pathological aspects [2]. Both diseases are assumed to be mediated by myelin-specific CD4^+ ^T lymphocytes, and more specifically by Th1 and Th17 cells. Although the relative contribution of each Th subset has not been clearly defined, there are reports of an encephalitogenic potential of both Th1 and Th17 cells and their signature cytokines, IFN-γ and IL-17, respectively [3,4].

Glucocorticoids (GC) are used to treat a wide range of inflammatory, allergic and autoimmune diseases, and they have been shown effective in the treatment of acute relapses in MS [5], as well as of EAE [6]. Multiple mechanisms are proposed to explain glucocorticoid therapeutic efficacy in autoimmune damage to the CNS [7], since GC down-regulate both innate and adaptive immune responses. Namely, GC have been shown to inhibit lymphocyte proliferation and lymphocyte expression and production of various pro-inflammatory cytokines and mediators (e.g. IL-1β, IL-6, TNF-α), while enhancing the expression of anti-inflammatory cytokines (e.g. IL-10, TGF-β), T-cell apoptosis and redistribution, a shift in the population of Th cells from Th1 to Th2 [8,9] and the proportion of regulatory cells [10]. We have recently demonstrated that methylprednisolone (MP), a synthetic glucocorticoid, inhibits the *in vitro *expression and production of IL-17 in myelin basic protein (MBP)-stimulated draining lymph node cells (DLNC) and cells infiltrating CNS of EAE rats [11].

Although abundant data about diverse glucocorticoid effects have been accumulated, the mechanisms underlying the beneficial effects of glucocorticoids and the major site of their action relevant to therapeutic efficiency in T cell-mediated CNS autoimmune diseases, such as MS and EAE, are not fully understood. Recent evidence from EAE induced in C57BL/6 mice suggests that the major targets of GC action are peripheral rather than CNS-residing T lymphocytes [12]. On the contrary, plentiful data convincingly demonstrate that GC directly influences cells within the target tissue [reviewed in [13]]. Therefore, in this study we investigated the effect of the synthetic glucocorticoid methylprednisolone (MP), on the expression and production of IFN-γ and IL-17 by T lymphocytes infiltrating CNS tissue in a rat model of EAE. We found that MP applied *in vivo *inhibited IFN-γ and IL-17 generation by the cells infiltrating the CNS. This inhibition correlated with a reduction of clinical signs of EAE in MP-treated animals, thus suggesting that the inhibition of IFN-γ and IL-17 within the CNS contributes to the beneficial effects of glucocorticoids in neuroinflammatory diseases.

## Methods

### Experimental animals and EAE induction

Inbred Dark Agouti (DA) rats were obtained from the animal breeding facility of the Institute for Biological Research "Siniša Stanković" (Belgrade). Age- and gender-matched animals, between 12 and 16 weeks of age, were used in the experiments. They were housed under conventional conditions with laboratory chow and water *ad libitum*. DA rats were immunized by intradermal injection in the hind footpad of 100 μl of emulsion made by a mixture of equal volumes of rat spinal cord homogenate (SCH) in phosphate-buffered saline (PBS) (50% w/v) and complete Freund's adjuvant (CFA; Difco Laboratories, Detroit, MI), as described previously [14]. Animals were monitored daily for clinical signs of EAE, and scored according to the following scale: 0, no clinical signs; 1, flaccid tail; 2, hind limb paresis; 3, hind limb paralysis; 4, moribund state or death.

### Treatment with MP and RU486

Beginning on the day when the first neurological signs appeared (designated as day 1) DA rats were injected daily for 3 days with methylprednisolone (MP, 50 mg/kg body weight, Hemofarm, Vršac, Serbia) or with MP+RU486 (25 mg/kg, Sigma-Aldrich, St. Louis, MO) or with PBS. Three hours after the last injection the animals were sacrificed and extensively perfused with cold PBS through the left ventricle. All animal experiments were carried out on the basis of respective protocols approved by the University of Belgrade School of Medicine Animal Ethics Committee (no 1320/1-3) and in accordance with the U.K. Animals (Scientific Procedures) Act, 1986 and associated guidelines, the European Communities Council Directive of 24 November 1986 (86/609/EEC).

### Isolation of lymph node cells, mononuclear cells from spinal cords and splenocytes

Draining (popliteal) lymph nodes (DLN) and spleens (SP) were isolated from immunized rats and cervical lymph nodes (CLN) were obtained from healthy animals. The lymph nodes and spleens were passed through a steel mesh in RPMI-1640 medium (Sigma-Aldrich, St Louis, MO), and the obtained suspensions of cells (DLNC, CLNC and SPC) were filtered through a nylon mesh, washed and resuspended in RPMI with 5% fetal calf serum (FCS) (PAA Laboratories, Pasching, Austria). Mononuclear cells were isolated from the spinal cords of individual immunized rats. Spinal cords were dissociated through a wire mesh and centrifuged at 700 g for 2 min. The pellets were then resuspended in 40% Percoll (Sigma-Aldrich) and centrifuged on a discontinuous 40%:70% Percoll gradient at 850 g for 55 min. The spinal cord mononuclear cells (SCC) were collected from the 40%/70% interface, two times washed (900 g for 5 min) in RPMI supplemented with 5% FCS, and resuspended in RPMI 5% FCS. SCC, DLNC and CLNC were grown at 5% CO2 and 37°C in RPMI-1640 supplemented with antibiotics. After the isolation all the cells were counted in 0,2% trypan blue solution using a hemocytometer. Peripheral blood (PB) was isolated from the hearts of immunized rats, mixed with Turk solution (10 μl of blood in 90 μl of Turk solution) and the number of cells (PBC) was counted using a hemocytometer.

### Reverse transcription - real time polymerase chain reaction

In order to determine cytokine gene expression, real time PCR was performed. Total RNA was isolated from the cells immediately after isolation from spinal cords using mi-Total RNA Isolation Kit (Metabion, Martinsried, Germany), and reverse-transcribed using random hexamer primers and MMLV (Moloney Murine Leukemia Virus) reverse transcriptase, according to manufacturer's instructions (Fermentas, Vilnius, Lithuania). Prepared cDNAs were amplified using Power SYBR^® ^Green PCR Master Mix (Applied Biosystems, Foster City, CA), according to the recommendations of the manufacturer, in a total volume of 20 μl in an ABI PRISM 7000 Sequence Detection System (Applied Biosystems). Thermocycler conditions comprised an initial step at 50°C for 5 minutes, followed by a step at 95°C for 10 minutes and a subsequent 2-step PCR program at 95°C for 15 seconds and 60°C for 60 seconds for 40 cycles. The PCR primers (Metabion) were as follows: IFN-γ forward primer 5'-TGG CAT AGA TGT GGA AGA AAA GAG-3'; IFN-γ- reverse primer 5'-TGC AGG ATT TTC ATG TCA CCA T-3'; IL-17 forward primer 5'-ATC AGG ACG CGC AAA CAT G-3'; IL-17 reverse primer 5'-TGA TCG CTG CTG CCT TCA C-3'; IL-23 forward primer 5'-TCC ACC AAA CTC CCC AGA CA-3'; IL-23R reverse primer 5'-CTG TGC ATG CTC TTT GGT TGA T-3'; RorG forward primer 5'-GAC AGG GCC CCA CAG AGA-3'; RorG reverse primer 5'-TTT GTG AGG TGT GGG TCT TCT TT-3'; T-Bet forward primer 5'-CCA ACA ATG TGA CCC AGA TGA T-3'; T-bet reverse primer 5'-CTG GCT CAC CGT CAT TCA-3'; β-actin forward primer 5'-GCT TCT TTG CAG CTC CTT CGT-3'; β- actin reverse primer 5'-CCA GCG CAG CGA TAT CG-3'. Accumulation of PCR products was detected in real time and the results were analyzed with 7500 System Software (AB) and presented as 2^-dCt^, where dCt was difference between Ct values of specific gene and endogenous control (β-actin).

### Cell-surface and intracellular staining and flow cytometric analysis

Anti-CD4-PE, anti-CD62L-biotin, OX40-biotin (all from BD Biosciences, San Jose, CA) anti-CD11b-FITC, anti-CD25-FITC (both from AbD Serotec, Oxford, UK) were used for phenotype analysis of CNS-infiltrating cells. Biotin-coupled antibodies were used in conjuction with SAv-FITC Conjugate (BD Biosciences). Annexin V coupled with FITC (Biotium Inc, Hayward, CA) was used for detection of apoptotic cells. Simultaneous detection of IFN-γ- and IL-17-producing cells was performed using intracellular staining after a short stimulation (4 h) with phorbol myristate acetate (PMA) (100 ng/ml) and ionomycin (400 ng/ml) in the presence of Brefeldin A (5 μM) (all from Sigma-Aldrich). First, cells were stained for CD4 by incubation with anti-CD4-PE antibody for 30 minutes, at +4°C. Subsequently, the cells were fixed with 4% paraformaldehyde (PFA) for 20 min at room temperature (RT). Cells were then stained with PE or FITC conjugated anti mouse-IL-17 and corresponding isotype control (BD Biosciences) and biotin conjugated anti-rat IFN-γ and corresponding isotype control (Biosource, Carlsbad, CA), followed by coupling with SAv-PerCP Conjugate (BD Biosciences). All the incubations were performed in permeabilization buffer (PBS, FCS 2%, BSA 0.1%, Triton X-100 0.1%). The stained cells were acquired by a FACSCalibur cytometer (BD Biosciences) and analyzed using CellQuest Software (BD Biosciences).

### ELISA

For determination of cytokine production, SCC were seeded in 96-well plates (5 × 10^5^/200 μl) and DLNC were seeded in 24-well plates (2.5 × 10^6^/1 ml) for 72 hours. SCC were grown in medium alone, and DLNC and CLNC in medium with concanavalin A (ConA, Sigma-Aldrich, 1 μg/ml). CLNC were cultivated in the presence of MP (10 ng/ml) with or without RU486 (5 ng/ml) for 24 hours. Subsequently, cell culture supernatants were collected and cells pelleted by centrifugation (5000 g, 3 min). Cell-free supernatants were frozen until they were analyzed on the basis of the protocol recommended by manufacturers of the ELISA kits (OptEIA Mouse IL-17 Set, BD Biosciences; Rat IFN-γ Set, R&D systems, Minneapolis, MI).

### Statistical analysis

The results are presented as mean ± SD of data obtained from at least three separate experiments with similar data. Student's t test was performed for statistical analysis. A *p *value of less than 0.05 was considered statistically significant.

## Results

### MP ameliorates EAE in DA rats and reduces infiltration of encephalitogenic cells into the CNS

**Figure 1:**
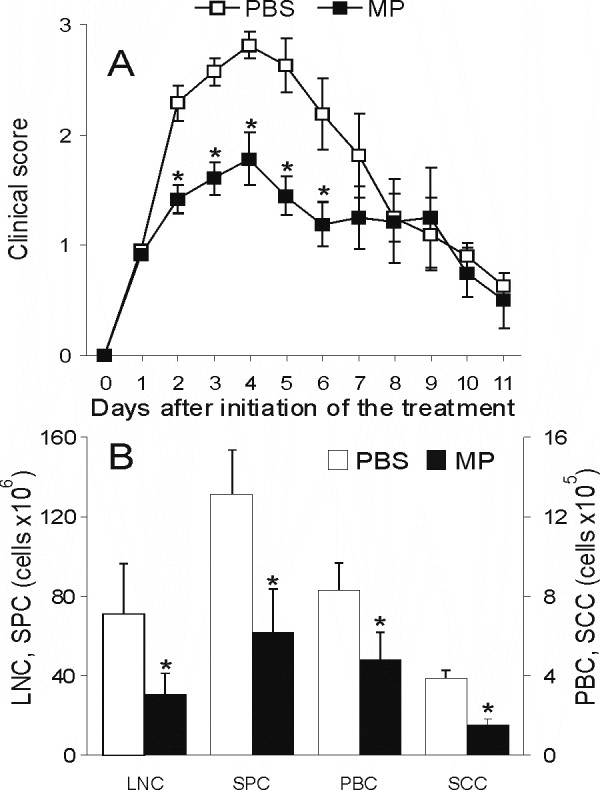
**MP treatment ameliorates EAE in DA rats and decreases infiltration of cells into the CNS**. DA rats were immunized with SCH-CFA. Commencing on the day when first neurological signs appeared (designated as day 1), DA rats were injected daily for 3 days with methylprednisolone (MP, 50 mg/kg body weight) or with phosphate-buffered saline (PBS). The rats were checked daily for clinical signs of EAE. For the determination of the clinical course of EAE, 19 animals per group (out of four independent experiments) were used. (A). Three hours after the last injection of MP some animals were sacrificed, and cells infiltrating spinal cord, splenocytes, peripheral blood cells, and draining lymph node cells were isolated and counted (B). *p < 0.05 represents statistically significant difference between MP and PBS group.

Rats were immunized with SCH-CFA and treated with MP (50 mg/kg) for three consecutive days following the appearance of initial clinical signs of EAE, to mimic steroid pulse therapy in MS. Control rats, treated with vehicle (PBS), were paired to MP-treated rats both according to the severity of initial clinical signs and to the time of the initial signs occurrence. In the group of control rats EAE progressed more rapidly than in MP-treated rats (Fig. 1A). Further, some rats from both MP-treated and control groups were sacrificed and cells infiltrating spinal cord (SCC) isolated for further analysis. Splenocytes (SPC), peripheral blood cells (PBC) and draining lymph node cells (DLNC) were also isolated from these rats. The number of cells isolated from all of the tissues of the two groups of rats sacrificed after 3-days of treatment differed significantly, with lower numbers detected in MP-treated rats (Fig 1B). The remaining rats were left for observation after cessation of MP treatment, and it appeared that MP-treated rats exhibited significantly milder disease compared to controls (Fig 1A). No relapses were observed in either group.

### MP inhibits IFN-γ and IL-17 expression and production in cells infiltrating CNS of EAE DA rats

**Figure 2:**
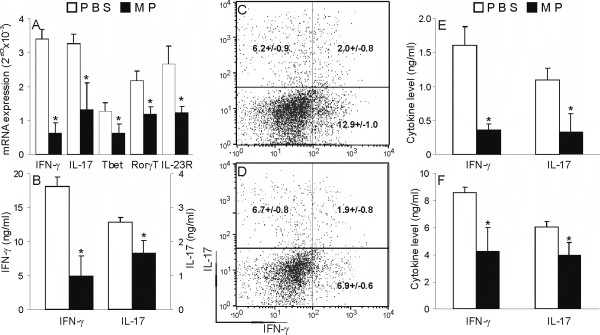
**MP inhibits IFN-γ, but not IL-17 production, in cells infiltrating the CNS of DA rats with EAE**. SCC and DLNC were isolated from DA rats immunized with SCH-CFA and injected with MP or PBS, as described in Methods. RNA was isolated from SCC and RT-PCR was performed (A), or SCC were grown in medium and ELISA was performed from cell-free supernatants obtained after 72 hours of cultivation (B). Alternatively, SCC from PBS-treated (C) and MP-treated (D) rats were stained for IFN-γ and IL-17. Results of intracellular staining (C, D) are representative of three independent experiments. The numbers in the plots are means ± SD of the proportions of cells present in the corresponding quadrant obtained in four independent experiments. DLNC were cultivated without (E) or with (F) 1 μg/ml of ConA for 72 hours and cell-culture supernatants were analyzed by ELISA. *p < 0.05 represents a statistically significant difference between the MP and PBS groups.

In order to explore the potential of MP to influence IFN-γ and IL-17 expression and production in cells infiltrating CNS, mononuclear cells isolated from spinal cord (SCC) were subjected to analyses of direct *ex vivo *RNA expression and production, as well as PMA+ionomycin-stimulated intracellular expression of IFN-γ and IL-17. MP applied *in vivo *had a clear-cut inhibitory effect on IFN-γ and IL-17 gene expression in SCC (Fig 2A), as well as on spontaneous release of cytokines from these cells (Fig 2B). Also, MP applied *in vivo *inhibited expression of the main Th1 and Th17 transcription factors, Tbet and ROR-γT, respectively, as well as of one of the markers of Th17 cells, IL-23R, in SCC (2A). Interestingly, if SCC were stimulated with PMA and ionomycin there were fewer cells expressing IFN-γ, but not IL-17, among SCC isolated from MP-treated rats in comparison to vehicle-treated rats (Fig 2C, D). SCC capable of expressing both IFN-γ and IL-17 were also observed and their percentage was moderately reduced under the influence of MP (Fig 2C, D). Thus, these results show that MP interferes with IFN-γ and IL-17 production in cells infiltrating CNS during EAE. Similarly, treatment of EAE rats with MP also affected spontaneous and ConA-stimulated release of IFN-γ and IL-17 from DLNC (Fig 2E, F).

### MP does not influence the proportion of CD4^+ ^cells among cells infiltrating CNS

**Figure 3:**
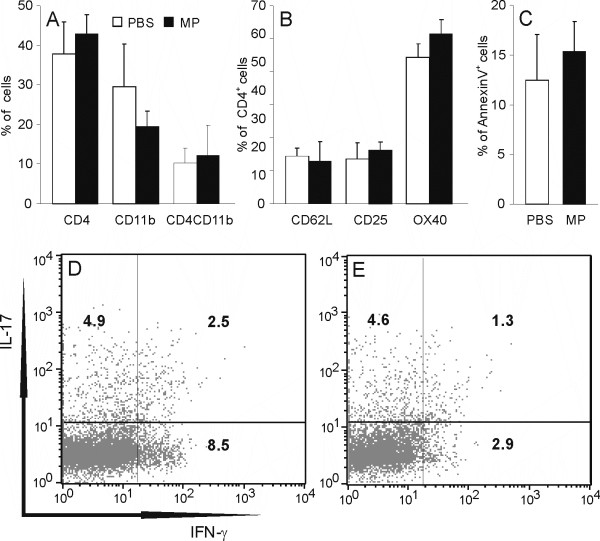
**MP does not affect CD4^+ ^cell proportion or apoptosis rate among CNS-infiltrating cells**. SCC were isolated from DA rats immunized with SCH-CFA, and injected with MP or with PBS, as described in Methods. SCC were stained with anti-CD4 and anti-CD11b specific antibodies (A), with anti-CD4 and anti-CD62L or anti-OX40 or anti-CD25 specific antibodies (B), or with anti-CD4 specific antibody and Annexin V (C). Alternatively, SCC from PBS-treated (D) and MP-treated (E) rats were stained with anti-CD4 antibody and intracellular staining for IFN-γ and IL-17 was performed. The results presented in B-E are gated to CD4^+ ^cells. Results of intracellular staining (D, E) are representative of three independent experiments. *p < 0.05 represents a statistically significant difference between the MP and PBS groups.

In order to find out if the observed effect of MP on IFN-γ and IL-17 expression is related to a change in proportion of CD4^+ ^lymphocytes as main producers of these cytokines among cells infiltrating CNS, and to explore whether apoptosis is induced in these cells under the influence of MP, cells isolated from spinal cords of MP- or PBS-treated rats were stained with labeled anti-CD4 and anti-CD11b antibodies, or with labeled anti-CD4 antibody and AnnexinV, and subjected to cytofluorimetric analysis. Among cells infiltrating CNS there were populations of CD4+ T cells (CD4^+bright^CD11b^-^), of macrophages/monocytes (CD4^+dim^CD11b^+ ^or CD4^-^CD11b^+^) and of other cell types (CD4^-^CD11b^-^) (Fig 3A). The percentage of CD4^+ ^T cells did not differ between MP-treated and control animals (Fig 3A). Moreover, the percentage of CD62L^+ ^(marker of naïve T cells), CD25^+ ^(activation marker of T cells) and OX40^+ ^(marker of activated T cells) cells was similar between MP-treated and control animals (Fig 3B). In addition, the proportion of AnnexinV+ (apoptotic) cells among CD4^+ ^cells was determined, and no difference was found between the MP-treated and control groups (Fig 3C). Thus, we could conclude that the observed effect of MP *in vivo *treatment was not mediated through modulation of the proportion of CD4^+ ^T cells among cells infiltrating the CNS. We could also conclude that MP did not alter the proportions of naïve and effector cells within the CD4^+ ^T cell population. Importantly, the difference in the effects of MP on IFN-γ and IL-17 in PMA+ionomycin-stimulated SCC was also evident if analyses of intracellular cytokine expression were restricted to CD4^+ ^cells (Fig 3D, E), thus suggesting that MP down-regulated IFN-γ, but not IL-17, in CNS-infiltrating Th populations stimulated with PMA and ionomycin.

### RU486 prevents downregulatory effects of MP on IFN-γ and IL-17 production by CNS-infiltrating cells

**Figure 4:**
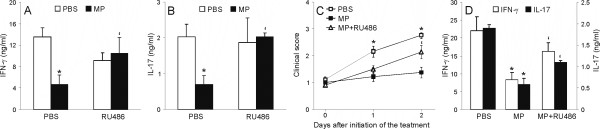
**RU486 prevents inhibition of IFN-γ and IL-17 by MP**. (A) DA rats were immunized with SCH-CFA. Commencing on the day when first neurological signs appeared (designated as day 1) DA rats were injected daily for 3 days with methylprednisolone (MP, 50 mg/kg body weight) and/or RU486 (25 mg/kg body weight) or with phosphate-buffered saline (PBS). The rats were checked daily for clinical signs of EAE. For the determination of the clinical course of EAE, 15 animals per group (out of three independent experiments) were used. (B, C) CLNC from healthy animals were stimulated with ConA (1 μg/ml) in the presence or absence of MP (10 ng/ml) and/or RU486 (5 ng/ml) for 24 hours. Cell-free supernatants of the cultures were analyzed with ELISA for IFN-γ (B) and IL-17 (C) concentrations. (D) SCC were isolated from DA rats immunized with SCH-CFA and injected with PBS, or with MP, or with MP+RU486, as described in Methods. Cell-free supernatants of cultures of SCC grown in medium for 72 hours were analyzed with ELISA for IFN-γ and IL-17 concentrations. *p < 0.05 represents a statistically significant difference between the MP and PBS treatment groups, and 'p < 0.05 represents a statistically significant difference between the MP and MP+RU486 treatment groups.

Further, we explored if the inhibitory effect of MP on IFN-γ and IL-17 production is dependent on glucocorticoid receptor engagement. Thus, we used a well-recognized glucocorticoid receptor antagonist RU486 [6]. First, we applied this agent *in vitro*, where cervical lymph node cells from healthy rats were stimulated with ConA in the presence or absence of MP and/or RU486. As a result, it was clear that RU486 antagonized the inhibition imposed upon ConA-stimulated CLNC by the influence of MP (Fig 4A, B). In order to investigate if the observed inhibition of IFN-γ and IL-17 production by SCC after *in vivo *MP treatment is mediated through glucocorticoid receptor-dependent mechanisms, DA rats were immunized with SCH-CFA and treated either with MP (50 mg/kg) or with MP (50 mg/kg) and RU486 (25 mg/kg) for three consecutive days following appearance of initial clinical signs of EAE. Control rats, treated with vehicle (PBS), MP-treated rats and MP+RU486-treated rats were paired both according to the severity of initial clinical signs and to the time of the initial signs occurrence. Importantly, RU486 antagonized the effect of MP on EAE clinical course (Fig 4C), as well as its effect on IFN-γ and IL-17 production by SCC (Fig 4D). Thus, these results imply that the inhibitory effect of MP on IFN-γ and IL-17 production both *in vitro *and *in vivo *is mediated through the glucocorticoid receptor.

## Discussion

This study demonstrates that MP given *in vivo *to DA rats with first signs of EAE, by a regimen that leads to significant amelioration of the disease, inhibits the expression and production of IFN-γ and IL-17 by CNS-infiltrating cells, thus suggesting that the beneficial effect of MP in EAE is partly mediated through restraining immune responses within the CNS. Limitation of production of IFN-γ and IL-17 in the CNS is of importance for the beneficial effects of MP, as abundant data confirm the pathogenic role for IFN-γ and IL-17 in autoimmune diseases, although their contribution to the disease process probably depend on the epitope-specificity of encephalitogenic cells [3], localization of the disease process [4] and conditions present during initial exposure to antigen, including the quality/quantity of Toll-like receptor stimulation and type of antigen-presenting cells [15]. In the EAE model in DA rats used in this study, it appears that both Th1 and Th17 cells with their marker cytokines play a role in the disease process [4].

The effects of glucocorticoids on immune cells can be mediated through glucocorticoid receptor-dependent and independent actions [16]. In fact, glucocorticoids bring about the majority of their effects upon interaction with a cytoplasmic glucocorticoid receptor (GR) which, subsequent to ligand binding, dissociates from chaperone proteins, forms dimers, and translocates into the nucleus where it modulates gene expression either by direct binding to its cognate response elements or via interaction with other transcription factors [17]. Recently, a new concept of non-genomic actions of glucocorticoids emerged which assumes that activated glucocorticoid receptor may produce effects without an action on gene transcription [reviewed in [18]]. Moreover, some effects of glucocorticoids may also be exerted without involvement of a GR; they are mediated by membrane-bound GRs, or as a result of direct, nonspecific interactions with cell membranes [18]. Therefore, we analyzed the effects of RU486, an antagonist of glucocorticoid receptor [6], in our experimental system and showed that this drug counteracted MP in its effect on EAE course, as well as in its *in vitro *and *in vivo *effects on IFN-γ and IL-17 generation. These results exclude the possibility that the effect of MP might result from its binding to membrane receptors or subcellular structures other than the glucocorticoid receptor. Moreover, since RU486 prevents the translocation of the glucocrticoid-GR complex to the nucleus [19], these results confirm that the observed inhibition of IFN-γ and IL-17 represents a genomic effect of MP.

In this study MP treatment commencing with the first clinical signs of actively induced EAE was applied to mimic application of the drug in acute relapses of MS in humans. In accordance with previously published results showing beneficial effects of GC treatment on EAE [8], MP treatment led to a significant amelioration of clinical signs of EAE in DA rats. The inhibitory effect of MP in this model of EAE was maintained throughout the follow-up evaluation period, 8 days after methylprednisolone withdrawal, with no relapse of disease. This observation is in agreement with a previously published report in which the authors applied a similar concentration of dexamethasone by the same application regime in active EAE in C57BL/6 mice [12]. However, if glucocorticoids are applied in lower concentration [20], or they are applied *per os *[10] or they are applied for a longer time [21] and then withdrawn, worsening of clinical features, reappearance of the CNS inflammatory infiltrate and lymphocyte reactivity to the encephalitogen are reported. Thus, it seems that the mode of glucocorticoid application is of utmost importance for their effect on EAE.

Multiple mechanisms have been postulated to be responsible for the beneficial effect of glucocorticoids on CNS autoimmunity [7], while peripheral T lymphocytes have been considered as the main therapeutic target [12]. However, an extensive literature demonstrates that glucocorticoids are effective in reducing CNS inflammation by direct action on cells within the CNS [reviewed in [13]]. Here, we show that the number of cells isolated from the spinal cord of MP-treated animals was significantly lower than the number of cells isolated from control rats in EAE. This could be a consequence of the inhibition of encephalitogenic cell infiltration into the CNS that has been previously reported following treatment of EAE with glucocorticoids [10]. The limitation of the infiltration might be explained by increased apoptosis and weaker activation of encephalitogenic cells in lymphoid organs, by decrease in expression of adhesion molecules on leukocytes and endothelial cells of the blood brain barrier (BBB), as well as by increased integrity of BBB [22,23,8]. On the other hand, fewer infiltrating cells within the CNS of MP-treated rats might also be a consequence of increased rate of apoptosis of these cells in the CNS, as previously suggested [24]. However, we could not detect increased cell death rate under the influence of MP in EAE rats, which is in accordance with a recent investigation performed in mice [12]. We have to emphasize that there is a possibility that a number of apoptotic cells was lost in the procedure of cell isolation, and that in vivo numbers of apoptotic cells in MP-treated animals might be higher within the CNS. However, this would still mean that among cells isolated from the CNS there is no difference in apoptosis regardless of the treatment with MP. The absence of increased apoptosis within the CNS of MP-treated rats might be explained by the dual death-related influence of glucocorticoids on T cells, as glucocorticoids induce apoptotic death of T cells but also protect them against CD95-mediated apoptosis, including activation-induced cell death, probably due to suppression of FasL expression [25] and/or an increase in Bcl-2 expression [26]. Still, the number of immune cells was down-regulated across various peripheral lymphoid tissues and blood under the influence of MP, and it seems reasonable to assume that apoptosis of these cells is at least partly responsible for the effect of MP. Apoptosis in the periphery could also be one of the major reasons for the decreased number of cells infiltrating the CNS.

In addition to the limitation of immune cells infiltration in the CNS, the restriction of pro-inflammatory potential of infiltrated cells could also be of importance for the beneficial effects of MP in EAE. Indeed, if equivalent numbers of spinal cord-infiltrating cells from MP-treated and control rats are compared, a significant difference in IFN-γ and IL-17 expression and production is observed. Thus, besides diminishing the number of infiltrating cells within the CNS, MP also restrains their pro-inflammatory i.e. autoimmune potential. As the major producers of these cytokines in the CNS of EAE rats are CD4^+ ^cells, the downregulation of cytokine production and expression in SCC might be a consequence of a reduced CD4^+ ^cell proportion among CNS-infiltrating cells. However, again in agreement with the findings of Wüst and colleagues [12], we do not find a difference in the percentage of CD4^+ ^cells among CNS-infiltrating cells. Moreover, there is no difference in the proportion of naive and activated cells within the population of CD4^+ ^cells, thus excluding the possibility that MP affects IFN-γ and IL-17 production through specific reduction of activated CD4^+ ^cells among CNS infiltrating cells.

While a large body of evidence shows that glucocorticoids inhibit Th1 and IFN-γ in EAE [reviewed in [27]], the influence of glucocorticoids on Th17 and their signature cytokine IL-17 has not been investigated in detail so far. We have recently shown that MP inhibits IL-17 *in vitro*, but to a lesser extent than it influences IFN-γ [11], and to that we add the similar observation from this study about IL-17 inhibition in the CNS of MP-treated rats. The eventual importance of other cytokines, or other regions which could also be affected, is not excluded.

Interestingly, when SCC were stimulated with PMA+ionomycin and intracellular detection of IFN-γ and IL-17 was performed, the proportion of IFN-γ-producing, but not of IL-17-producing cells, was reduced in cells isolated from MP-treated rats in comparison to control rats. The proportion of these cells that are capable of expressing both cytokines, and that have recently been described in DA rat EAE [14], was also not affected by MP treatment, thus suggesting that these cells are more similar to Th17 than to Th1, at least regarding sensitivity to glucocorticoid regulation. This implies that although MP applied *in vivo *restrains generation capacity of both cytokines within the CNS, SCC released from the CNS environment and stimulated with strong unspecific stimulus regain the potential to produce IL-17, but not IFN-γ. Importantly, the same observation was valid for SCC in general and for CD4^+ ^cells i.e. Th1 and Th17 cells. Thus, it seems that after PMA+ionomycin treatment, spinal cord-derived T cells display an apparent shift from Th1 to Th17 cells Although we do not yet have an explanation for these findings, it is tempting to speculate that the influence of MP on Th1 might be direct, while the drug might affect Th17 cells and their cytokines through its effect on CNS resident cells, which have previously been shown to modulate IL-17 production at least *in vitro *[28,29]. Alternatively, there might be a difference in the duration of the inhibitory effect of MP on Th1 and Th17 cells, and stimulation with PMA and ionomycin takes 4 hours, which is enough time to express cytokines in lymphocytes. Also, there is a possibility that PMA+ionomycin, as a strong artificial treatment, may skew the result through stimulation of both encephalitogenic and bystander cells. It remains for future investigations to determine the mechanism underlying the observed difference between *ex vivo *and post-activation IL-17 expression in SCC from MP-treated animals. It would also be important to find out if this property of Th17 cells might be of relevance for the pathology of CNS neuroinflammation, especially regarding contribution of endogenous glucocorticoids to pathology resolution.

## Conclusions

Our data reveal a significant influence of MP on the expression and production of IFN-γ and IL-17 within the CNS in EAE. Thus, the beneficial effects of glucocorticoids in neuroinflammation are at least partly mediated through their ability to limit production of the inflammation-promoting cytokines IFN-γ and IL-17 in target tissue.

## List of abbreviation used

BBB: blood brain barrier; CFA: complete Freund's adjuvant; CLN: (cervical lymph nodes); CLNC: (cervical lymph node cells); CNS: central nervous system; ConA: concanavalin A; DA: dark agouti; DLN: draining lymph nodes; DLNC: draining lymph node cells; EAE: experimental autoimmune encephalomyelitis; FCS: fetal calf serum; GC: glucocorticoids; GR: glucocorticoid receptor; IFN: interferon; IL: interleukin; LNC: lymph node cells; MBP: myelin basic protein; MMLV: moloney murine leukemia virus; MP: methylprednisolone; MS: multiple sclerosis; PBC: perypheral blood cells; PBS: phosphate-buffered saline; PFA: paraformalehide; PMA: phorbol myristate acetate; SCC: spinal cord cells; SCH: spinal cord homogenate; SPC: spleen cells; TGF: transforming growth factor; Th: helper T cells; TNF: tumor necrosis factor.

## Competing interests

The authors declare that they have no competing interests.

## Authors' contributions

MM and ŽM carried out experimental procedures and helped to draft the manuscript; DM carried some of the experimental procedures, participated in its design and coordination and helped to draft the manuscript; MM-S conceived of the study, participated in its design and coordination, helped to draft the manuscript and carried some of the experimental procedures. All authors read and approved the final manuscript.
